# Optical Design of a Hyperspectral Remote-Sensing System Based on an Image-Slicer Integral Field Unit in the Short-Wave Infrared Band

**DOI:** 10.3390/s24124004

**Published:** 2024-06-20

**Authors:** Yi Ding, Chunyu Liu, Guoxiu Zhang, Pengfei Hao, Shuai Liu, Yingming Zhao, Yuxin Zhang, Hongxin Liu

**Affiliations:** 1 Changchun Institute of Optics, Fine Mechanics and Physics, Chinese Academy of Sciences, Changchun 130033, China; dingyi20@mails.ucas.ac.cn (Y.D.); liushuai@ciomp.ac.cn (S.L.); zhaoyingming20@mails.ucas.ac.cn (Y.Z.); zhangyuxin@ciomp.ac.cn (Y.Z.); liuhongxin22@mails.ucas.ac.cn (H.L.); 2 University of Chinese Academy of Sciences, Beijing 100049, China; 3 Research Center of the Satellite Technology, Harbin Institute of Technology, Harbin 150001, China; 18243087705@163.com; 4 The Military Representative Office in Changchun of Military Representative Bureau of Space System Equipment Department, Changchun 130033, China; haopengfeiwy@163.com

**Keywords:** image-slicer integral field unit, hyperspectral remote sensing, dynamic target spectral detection

## Abstract

Grating-type spectral imaging systems are frequently employed in scenes for high-resolution remote-sensing observations of the Earth. However, the entrance of the grating-type spectral imaging system is a slit or a pinhole. This structure relies on the push broom method, which presents a challenge in capturing spectral information of transiently changing targets. To address this issue, the IFU is used to slice the focal plane of the telescope system, thereby expanding the instantaneous field of view (IFOV) of the grating-type spectral imaging system. The aberrations introduced by the expansion of the single-slice field of view (FOV) of the IFU are corrected, and the conversion of the IFU’s FOV from arcseconds to degrees is achieved. The design of a spectral imaging system based on an image-slicer IFU for remote sensing is finally completed. The system has a wavelength range of 1400 nm to 2000 nm, and a spectral resolution of better than 3 nm. Compared with the traditional grating-type spectral imaging system, its IFOV is expanded by a factor of four. And it allows for the capture of complete spectral information of transiently changing targets through a single exposure. The simulation results demonstrate that the system has good performance at each sub-slit, thereby validating the effectiveness and advantages of the proposed system for dynamic target capture in remote sensing.

## 1. Introduction

Hyperspectral remote-sensing technology is capable of acquiring spatial and spectral three-dimensional data of targets [1,2], as well as analyzing targets’ structure and composition [3,4]. This technology offers significant advantages in target classification, identification and detection [5,6]. It is used in a variety of fields, including precision agriculture, military reconnaissance and mineral exploration [7,8].

Dispersive spectral imaging systems have been a focus in the field of spectral imaging due to their simple design [9]. Gratings are one of the commonly used optical elements in dispersive spectrometers, and they are used to achieve high spectral resolution due to their high dispersion efficiency [10]. Based on the principle of grating dispersion, the incident light source of a grating spectral imaging system is a slit or a pinhole. A single shot can only acquire single-line spectral information or single-point spectral information of the target [11,12,13]. Therefore, the grating spectral imaging system needs to sweep along the direction perpendicular to the slit to acquire the full spectral information of the target [14,15]. This method results in a reduction in temporal resolution due to the necessity of multiple exposures, which in turn limits the ability to capture the full spectral information of a target exhibiting transient changes. This restricts its application in dynamic scenes. Furthermore, the limitation of the pinhole or single slit also results in the waste of the telescope system’s FOV, which reduces the system’s overall efficiency.

The Integral Field Spectrometer (IFS) is capable of acquiring the full spectral information of a target in a single exposure without the need for a scanning component. This is achieved through the use of an IFU, which maps the spectral information from each sub-field of the telescope system onto the detector simultaneously [16]. The use of an IFU overcomes the limitations of traditional grating-type spectral imaging systems due to the introduction of pinholes or slits, and effectively avoids the system’s dependence on platform stability.

The image-slicer IFS offers a number of advantages, including continuous acquisition, three-dimensional spectral imaging, and high temporal resolution detection. It is a pivotal technology in the development of complex astronomical scientific instruments, as evidenced by the following references: [17,18,19,20]. At present, only a select number of typical astronomical telescopes are equipped with the image-slicer IFS [21,22,23,24,25]. The GNIRS (GEMINI near-infrared spectrograph) on the Gemini South telescope adopted the scheme of the image-slicer IFU, which realized low and medium spectral resolution detection in the wavelength range of 1–5 μm within a FOV of 3.2″ × 4.8″ [26,27]. The NIRSpec (Near-Infrared Spectrograph) equipped on the JWST (James Webb Space Telescope) also utilized an image-slicer IFU to achieve continuous sampling of a 3.1″ × 3.2″ sky region [28,29,30]. The MUSE (Multi Unit Spectroscopic Explorer) instrument, equipped on the VLT (second-generation Very Large Telescope), employed an image-slicer IFU for multi-object observations. This instrument offers two observation modes: a 1′ × 1′ FOV for wide-field observations and a 7.5″ × 7.5″ FOV for narrow-field observations [31,32]. In addition, the GIRMOS (infrared multi-target integrated field-of-view spectrometer) equipped on the Gemini North telescope, which utilized the image-slicer IFU to achieve the sample of the individual FOV of 1″ × 1″, 2″ × 2″ and 4″ × 4″ [33]. In astronomical observations, the image-slicer IFU is primarily concerned with the sampling of extended objects or multi-object observations of point-like objects. The FOV of such instruments is typically in the range of a few arcseconds [34]. However, the FOV of spectral imaging systems used for Earth remote sensing is usually expressed in angular terms. Increasing the FOV will result in large optical aberrations [35]. Although the image-slicer IFUs have the capacity to expand the IFOV of spectral imaging systems, the significant discrepancy between the single slice’s FOV in astronomy (arcseconds) and Earth remote sensing (degrees) limits the applicability of the image-slicer IFU in Earth remote sensing spectral imaging systems. Consequently, it remains a great challenge to apply the image-slicer IFU to spectral imaging systems in the field of Earth remote sensing to realize the expansion of its IFOV.

In this paper, a spectral imaging system based on an image-slicer used in short-wave infrared is proposed for the field of Earth remote sensing. The system employs the image-slicer IFU to slice the focal plane of the telescope system and integrate it into the form of virtual slit array, thereby achieving the expansion of the IFOV. It adopts a step-by-step magnification reduction approach to achieve the low F-number requirement of hyperspectral remote-sensing systems. This is accomplished by cumulating the magnification of each subsystem. Furthermore, freeform surfaces are incorporated into the spectral imaging system to effectively control the aberrations resulting from the increase in the full FOV of a single image slicer. This paper addresses the gap in the FOV of an image-slicer’s single slice between remote-sensing and astronomical fields, and presents a design method of an image-slicer IFU spectral imaging system applicable to remote sensing. And the system design and analysis are carried out according to a set of indicators. The expansion of the IFOV of a grating-type spectral imaging system is achieved by utilizing the image-slicer IFU as the core component. The design of an integrated optical system capable of capturing full spectral information of transiently changing targets is completed. The system provides an innovative solution to the challenge of the rapid detection of dynamic targets in the field of remote sensing. The layout of this paper is as follows: Section 2 presents the principle of a spectral imaging system based on an image-slicer IFU for the remote sensing of the Earth and describes the detailed specifications of this system. Section 3 outlines the design methodology employed in the design of the fore-optical system, the image-slicer IFU and the spectral imaging system, as well as the integration and optimization of the whole system. The methodology is subjected to simulation, with the results analyzed in detail in Section 4 to verify the effectiveness and feasibility of the design. Finally, a summary and discussion of the system are presented in Section 5.

## 2. Principle and System Description

### 2.1. Working Principle

**Figure 1:**
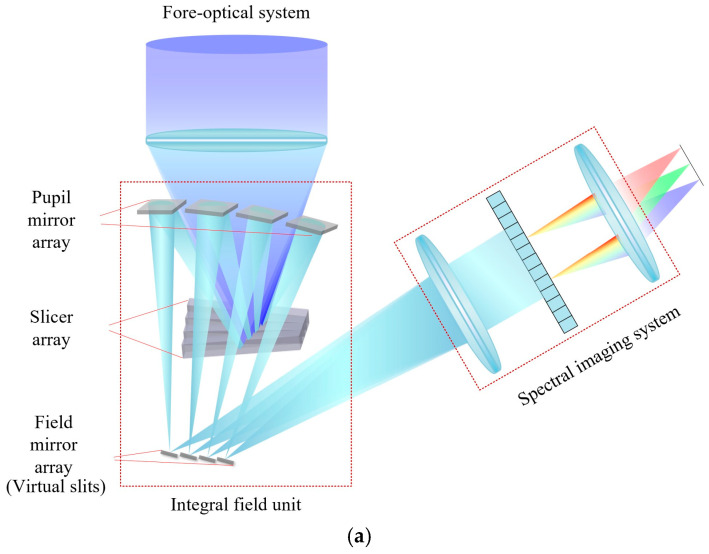

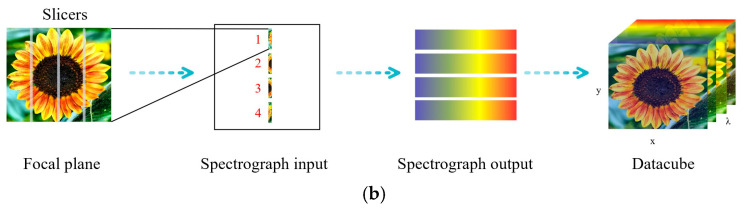
Principle of the image-slicer IFS: (**a**) schematic of the optical path structure of the system and (**b**) schematic of a 3D data cube formation from the focal plane of the fore-optical system.

The fundamental principle of the image-slicer IFS is illustrated in Figure 1 [18]. The IFS consists of three main subsystems: the fore-optical system, the image-slicer IFU, and the spectral imaging system. The image-slicer IFU is the key component of the system to realize the IFOV expansion. The image-slicer IFU is comprised of three arrays of mirrors: the slicer array, the pupil mirror array, and the field mirror array. The slicer array is located on the image plane of the fore-optical system, and uses the different two-dimensional tilt angles of each sub-mirror in the array to achieve image plane slicing and transmission. The pupil mirror corresponds to the slicer mirror one by one. By adjusting the tilt angles of the pupil mirrors, the slices can be reimaged and rearranged, thus completing the conversion of the target image to a virtual sub-slit array. The field mirrors are located in the virtual slit array and also correspond to the pupil mirrors. The system designed in this paper maintains the feature of a common pupil surface of the astronomical IFU. The co-planar of the exit pupil of the sub-slits can be achieved by setting the tilt angles for the field mirrors.

In this paper, we innovatively propose to apply the image-slicer IFU in remote-sensing spectral imaging systems. Compared with astronomical IFU, the IFU for a hyperspectral remote-sensing system has higher requirements for F-number and full FOV. In order to reduce the design difficulty at the IFU and further integrate the system, the fore-optical system was split into the telescope system and the relay system, which were used to deform and zoom the target image. Then, the F-number was accumulated by decreasing the magnification step by step. At the same time, the aberrations resulting from the expansion of the full FOV of the IFU’s single slice were corrected by means of a Zernike polynomial free-form surface. The system employs an ingenious design to slice, rearrange, and re-image the target image plane into a virtual slit array, which is then used as input to the spectral imaging system. The data cube corresponding to the three dimensions (x, y, λ) of the target point is obtained by reconstructing the spectral bands in each subfield of view, without the need for scanning, which reduces the requirements for platform stability. Furthermore, since the spectra of each spatial point are acquired simultaneously, the spectral information is stable with respect to time. This feature renders the system particularly well-suited to the acquisition of spectral information on dynamic targets.

### 2.2. System Description

**Figure 2:**
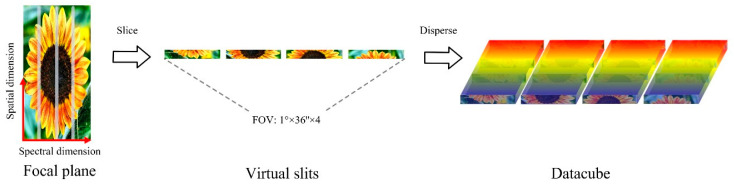
Schematic diagram of the field of view cut and its dispersion.

The relevant indicators and performance of the system designed in this paper are listed in Table 1. The system covers a spectral range of 1400 nm to 2000 nm, with a spatial resolution of 37.5 m and a spectral resolution better than 3 nm. The total FOV of the system is 1°, and the IFOV is larger than 0.5 mrad. As shown in Figure 2, the field of view in this system is sliced along the spectral dimension, and the field of view in the direction of the spatial dimension remains unchanged. Therefore, for a single slice whose spectral dimension corresponds to an IFOV of 36″ and the spatial dimension corresponds to a field of view of 1°, the field of view can be expressed as 1° × 36″.

The focal plane of the telescope system was sliced and linearly arranged using an image-slicer IFU, thereby completing the conversion from a two-dimensional image plane to a slit array. The entire slit array can be dispersed and mapped onto the detector by the same spectral imaging system, thus expanding the IFOV. This allows for the rapid capture of targets’ spectral information with transient changes in a single exposure. This paper presents a step-by-step design and combination optimization of the optical system, which is carried out according to the sampling requirements and the pupil-matching principle. This ensures that the system has good performance in any configuration.

## 3. Optical Design Process

### 3.1. Fore-Optical System

**Figure 3:**
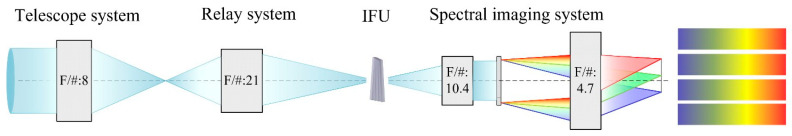
Schematic of a spectral imaging system based on an image-slicer IFU for remote sensing.

The fore-optical system is divided into two components: a telescope system and an anamorphic zoom relay system. The latter is employed to complete the matching between the former and the IFU. The telescope system is designed as a telecentric system in order to match the relay system. As shown in Figure 3, the design methodology of reducing the magnification of the whole system step by step is illustrated, where the labeling of the F-number indicates the F-number of the beam after it passes through the system. The image plane formed by the telescope system passes through the relay system, is magnified and deformed, and subsequently imaged on the slicers. The relay system serves two primary functions. Primarily, the relay system serves to provide an enlarged input image for the slicer. Secondly, the relay system is responsible for providing a deformation ratio to avoid oversampling in the spatial dimension [33]. In this system, the IFU slices the target image along the x-axis and arranges the sub-images along the y-axis. In the case of the spectral imaging system, the image is dispersed along the x-axis. A biconical plane is incorporated into the relay system to deform the target image, thereby maximizing the utilization of pixels while satisfying the Nyquist sampling criterion [36]. The biconical surface can be expressed as in Equation (1):

(1) $$Z=\frac{c_{x}x^{2}+c_{y}y^{2}}{1+\sqrt{1-\left(1+k_{x}\right)c_{x}^{2}x^{2}-\left(1+k_{y}\right)c_{y}^{2}y^{2}}}$$

where $c_{x}$ and $c_{y}$ are the curvatures of the surface in the x and y directions; and $k_{x}$ and $k_{y}$ are the corresponding conic constants. The distinction in the curvature of this surface shape in both the x and y directions allows for the generation of a deformed image with an x:y ratio of 2:1.

### 3.2. Image-Slicer IFU

**Figure 4:**
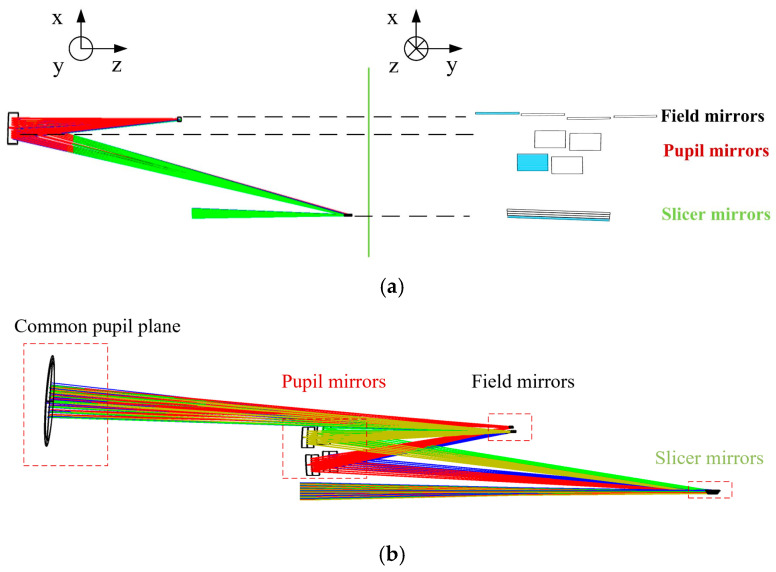
Optical path structure of the IFU: (**a**) schematic arrangement of the mirrors and (**b**) optical path of the system simulation.

The simplified model and optical path diagram of the IFU designed in this paper are shown in Figure 4 [37]. Figure 4a shows the single optical path (left) and geometric arrangement (right), which describe the optical path structure of the single configuration and the positional relationship and arrangement of the three mirror arrays in the IFU. The pupil mirrors are arranged in two rows and the field mirror positions are arranged in a single row. Figure 4b shows the three-dimensional optical path of the full configuration of the IFU in this paper, in which the slicer mirrors use cylindrical mirrors, and the pupil mirrors and field mirrors both use spherical mirrors. The IFU in this system is designed to perform three principal functions:

1. Focal plane rearrangement: the focal plane of the telescope system is sliced, rearranged and transmitted by slicer mirrors;
2. Providing a zoom ratio: the IFU uses the combination of slicer mirrors and pupil mirrors to produce a certain zoom effect on the sliced image in order to meet the F-number requirement of the back-end spectral imaging system design;
3. Common pupil design: the output of the IFU is a set of slit arrays. In terms of optical system design, it can be considered equivalent to a system comprising n (where n is the number of slits) sub-slits. To further integrate the system, the same spectral imaging system is used in this paper to perform dispersion imaging of the entire slit array. In order to create a common entrance for the individual sub-slits into the back-end spectral imaging system, the IFU needs to utilize the tilt of the field mirrors to create a common pupil plane.

**Figure 5:**
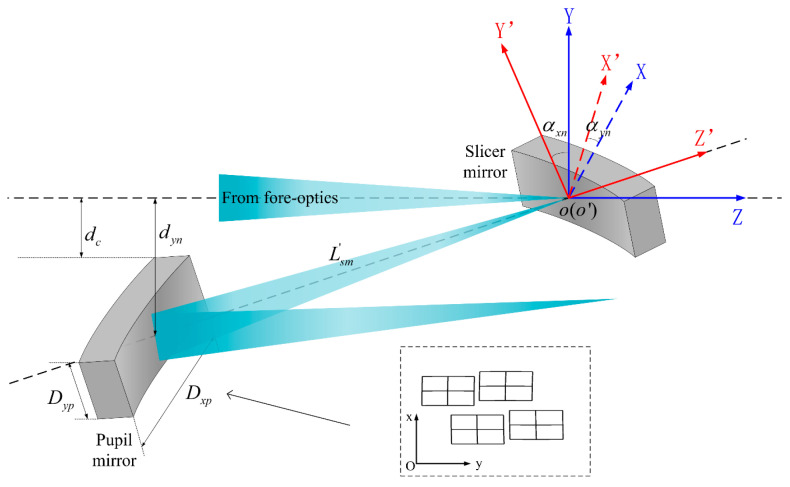
Schematic of the tilt of sub-mirrors within a single group IFU.

In the image-slicer IFU spectral imaging system, the slicing and rearranging of the targets’ image depend primarily on the tilt and arrangement relationships of the sub-mirror arrays within the IFU. The tilt angles of the slicers determine the position of the pupil mirrors. To avoid crosstalk between multiple configurations, the tilt angles of the slicers need to ensure the chief rays of each configuration are separated from the chief ray of fore-optics by a distance greater than half the diameter of the pupil mirror. The offset distance between the center of the pupil mirror and the main optical axis along the y-direction is determined by the tilt angle $\alpha_{xn}$; the offset distance between the centers of adjacent pupil mirrors along the x-direction is determined by the tilt angle $\alpha_{yn}$, where $\alpha_{xn}$ and $\alpha_{yn}$ represent the angle of rotation of the image slicers around both the x-axis and the y-axis. The pupil mirrors are arranged in rows and columns, as shown in Figure 5, where $\alpha_{xn}$ and $\alpha_{yn}$ are calculated in the same way. Taking $\alpha_{xn}$ as an example, we can obtain Equation (2):

(2) $$\alpha_{xn}=\frac{1}{2}\times \arcsin\frac{d_{yn}}{L_{sm}^{\prime }}$$

where $d_{yn}$ is the perpendicular distance from the center of the pupil mirror to the optical axis of the fore-optical system, and $L_{sm}^{\prime }$ is the distance between the slicer and the pupil mirror along the direction of light propagation. The imaging position of the exit pupil of the fore-optical system after passing through the slicer is the initial position of the pupil mirror. We assume that the distance between the slicer and the exit pupil of the fore-optical system is $L_{fep}$. The dimension of the exit pupil is $D_{xfep}$ in x-direction, and the dimension of the exit pupil is $D_{yfep}$ in y-direction. The dimension of the pupil mirror is $D_{yp}\times D_{xp}$. According to the relationship of objects and images, we can get Equation (3):

(3) $$L_{sm}^{\prime }=\frac{L_{fep}\times D_{yp}}{D_{fep}}$$

where $d_{yn}$ is determined by the arrangement of the pupil mirrors, and $d_{c}$ is the perpendicular distance from the upper edge of the pupil mirror to the optical axis of the fore-optical system. The position of the nth row of pupil mirrors, according to Equation (4), is as follows:

(4) $$d_{yn}=d_{c}+\frac{2n-1}{2}D_{yp}$$

Under certain constraints, the initial tilt angles $\alpha_{xn}$ and $\alpha_{yn}$ of the slicers can be calculated using the above formulas, and then the position of the pupil mirrors can be obtained using the object–image relationship so as to complete the initial arrangement of the IFU. Based on this, the design can be further adjusted to make the optical layout more reasonable.

### 3.3. Spectral Imaging System

The input to the spectral imaging system is provided by the virtual slit array generated by the IFU, and the IFOV expansion can be realized by simultaneously dispersing the virtual slit array and mapping it onto the detector. Taking the system designed in this paper as an example, its temporal resolution is improved by four times compared with the traditional spectral remote-sensing system, and even higher temporal resolution can be realized by further superimposing the number of slicers.

The IFU separates light by tilting the slicers. The larger the image square aperture angle of the telescope system, the larger the tilt angles required, and the larger the off-axis aberrations thus introduced. In order to reduce the difficulty of the IFU for use in remote sensing, a large F-number was retained at the IFU, and the F-number of the whole system was further decreased by the spectral imaging system with a smaller magnification in order to ensure the required resolution of the entire system. Based on the above description, we can obtain Equations (5) and (6):

(5) $$\beta =\beta_{1}\times \beta_{2}\times \beta_{3}$$

(6) $$F^{\#}=F_{fore}^{\#}\times \beta$$

where β is the magnification of the overall system, $\beta_{1}$ is the magnification of the relay system, $\beta_{2}$ is the magnification provided by the IFU, and $\beta_{3}$ is the magnification provided by the spectral imaging system. $F^{\#}$ and $F_{fore}^{\#}$ represent the F-number of the overall system and the telescope system, respectively. The above equations show that using a stepwise reduction in the magnification can reduce the design difficulty of the IFU without affecting the overall system indicators, and can also make the overall optical layout more reasonable.

Compared with the conventional long-slit spectrometers, the input to the spectral imaging system designed in this paper is a set of slit arrays with continuity. In order to use the same spectral imaging system for the simultaneous dispersion of the slit array, a common pupil plane is present at the output of the IFU, which serves as the sole entrance to the spectral imaging system.

The IFU designed in this paper is utilized in remote sensing with a full FOV of 1° for the single image slicer, while the corresponding FOV in the astronomical field is in the order of arcseconds, and the difference between the two is very large. The large increase in the full FOV of the single image slicer will result in some deviation in pupil overlap for different sub-slits. Therefore, this design incorporates a free-form surface in the spectral imaging system to increase the degrees of freedom for system optimization [38]. The surface is defined in terms of Zernike standard polynomials, and the surface sag follows Equation (7):

(7) $$Z=\frac{cr^{2}}{1+\sqrt{1-\left(1+k\right)c^{2}r^{2}}}+\overset{8}{\underset{i=1}{\sum }}\alpha_{i}r^{2i}+\overset{N}{\underset{i=1}{\sum }}A_{i}Z_{i}\left(\rho ,\varphi \right)$$

where c and k are the curvature and conic constant of the surface, r is the radial ray coordinate, N is the number of Zernike coefficients in the series, $A_{i}$ is the coefficient on the ith Zernike Standard polynomial, ρ is the normalized radial ray coordinate, and φ is the angular ray coordinate.

Increasing the number of terms in the polynomial will bring more free variables to the optimization of the system; the aberrations caused by the pupil deviation between the different sub-slits due to increasing the full FOV of the IFU can be corrected in the case of a limited number of lenses.

### 3.4. System Matching and Combined Optimization

After the design of the telescope system, the relay system, the image-slicer IFU, and the spectral imaging system is completed, the subsystems are combined according to the pupil-matching principle, and the whole system is integrated and optimized to further balance the aberrations. The integration optimization of the system in this paper focuses on the design issues due to the IFU technology migration. The use of a free-form surface is employed to balance the aberrations caused by pupil deviation between different configurations. Furthermore, additional adjustments have been made to the overall system to ensure that the residual aberrations remain within acceptable limits after integration and optimization.

## 4. Simulation and Analysis

**Figure 6:**
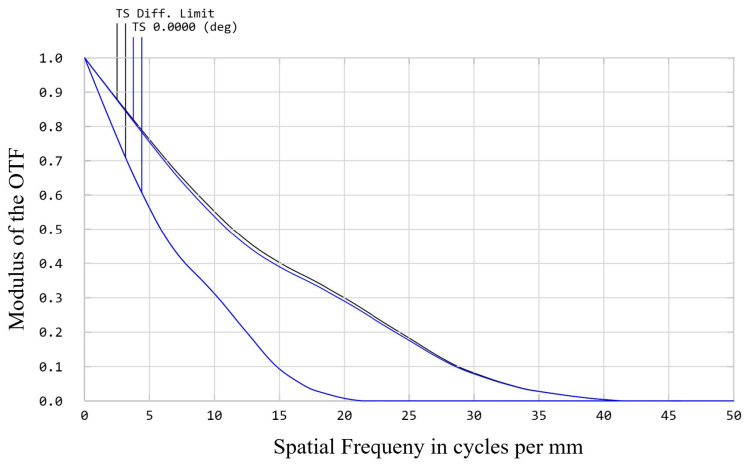
MTF curve of the fore-optical system.

To verify the effectiveness and feasibility of the above design, we designed the spectral imaging system based on the image-slicer IFU according to the indicators listed in Table 1. In this paper, a telescope system with an F-number of 8 is designed to image the target, which is then combined with an anamorphic and zoom relay system to provide the slicer with a magnified input image with a deformation ratio of 2:1. Figure 6 shows the modulation transfer function (MTF) curve of the fore-optical system. The blue line shows the MTF value for configuration 1 in the system, and the black line is the diffraction limit value. The difference between the values of the sagittal and the tangential is brought about by the deformation of the relay system.

In the design and simulation of the IFU, the tilt and offset of the internal sub-mirror arrays are primarily used to slice the two-dimensional image of the target and arrange it in the form of a continuous linear slit array. The initial tilt angles between the four configurations are calculated according to the formula in Section 3.2 and adjusted according to the actual optical path on this basis. The adjusted tilt angles of each sub-mirror array are shown in Table 2. In order to compensate for some of the off-axis aberrations of the system, the tilt angles of the slicers are compensated for by the tilt angles of the pupil mirrors. The tilt angles of the field mirrors are used to adjust the sub-pupils of the four configurations in such a way that they have a common entrance to enter the same spectral imaging system.

**Figure 7:**
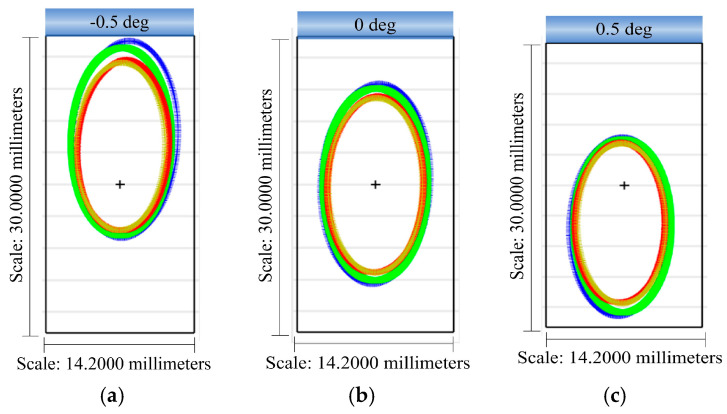
Footprint diagram of the common pupil: (**a**) footprint diagram of the common pupil plane at −0.5 deg; (**b**) footprint diagram of the common pupil plane at 0 deg; and (**c**) footprint diagram of the common pupil plane at 0.5 deg.

Figure 7 shows the footprint diagram at the common pupil plane. Figure 7a represents the center FOV, while Figure 7b,c illustrate the two edge FOVs, respectively. It can be observed that the pupils of the four configurations overlap almost exactly in the center FOV, but there is still some deviation in the edge FOV. This discrepancy has implications for the use of the same system for dispersion, so it is necessary to use the design of the back-end spectral imaging system for correction.

**Figure 8:**
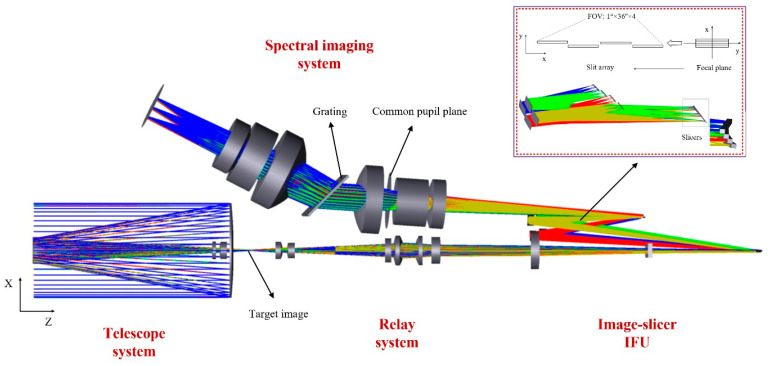
Optical design of the spectral imaging system based on an image-slicer IFU for remote sensing.

The structure of the integrated optical system is shown in Figure 8. The spectral imaging system includes a collimation system, a diffraction grating and an imaging system, which serves two functions. The first of these is to reduce the magnification of the system. In order to match with the IFU, the object square numerical aperture of the spectral imaging system is 0.048, and the cumulative F-number of the entire system is better than 4.7, which corresponds to the magnification of $\beta_{3}$ of approximately 0.45. The second objective is to correct the aberrations caused by the common pupil deviation between multiple configurations due to the expanded FOV. The 6th–12th of Zernike polynomial order is chosen for this system, and the simulation results show that this surface type can significantly improve the image quality of this system.

**Figure 9:**
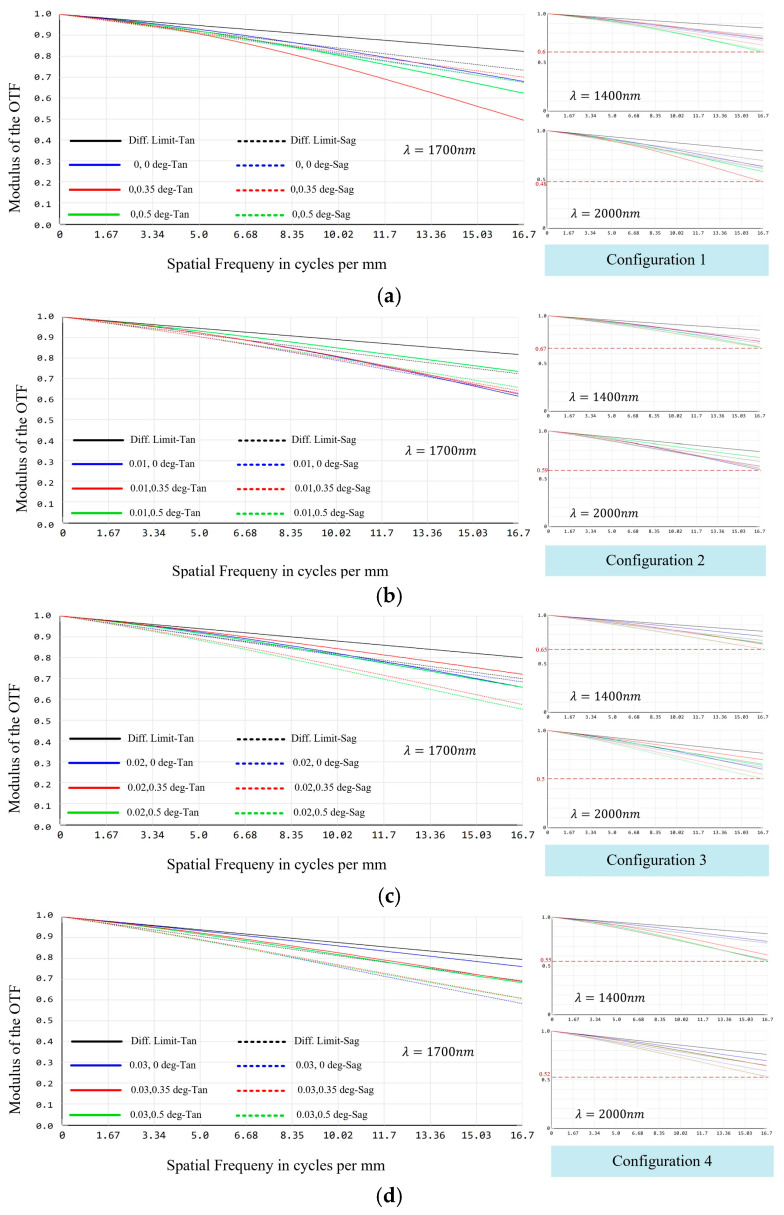
MTF curves of the spectral imaging system based on an image-slicer IFU: (**a**) MTF curves in configuration 1 for the wavelengths of 1700 nm, 1400 nm, and 2000 nm; (**b**) MTF curves in configuration 2 for the wavelengths of 1700 nm, 1400 nm, and 2000 nm; (**c**) MTF curves in configuration 3 for the wavelengths of 1700 nm, 1400 nm, and 2000 nm; and (**d**) MTF curves in configuration 4 for the wavelengths of 1700 nm, 1400 nm, and 2000 nm.

The planned methodology involves the utilization of a detector with a pixel size of 15 μm, with two pixels employed to sample the spectral dimension and one pixel to sample the spatial dimension. Figure 9 shows the MTF curves of the entire system. Compared with the conventional long-slit spectroscopic system, the system designed in this paper is equivalent to the simultaneous observation of four sets of slits. Consequently, the MTF at the center, maximum, and minimum wavelengths of the four sub-fields of view of the IFU need to be evaluated. The results demonstrate that the values of the MTF for the full field of view at 1400 nm, 1700 nm, and 2000 nm are better than 0.48 at 16.67 lp/mm for each configuration.

**Figure 10:**
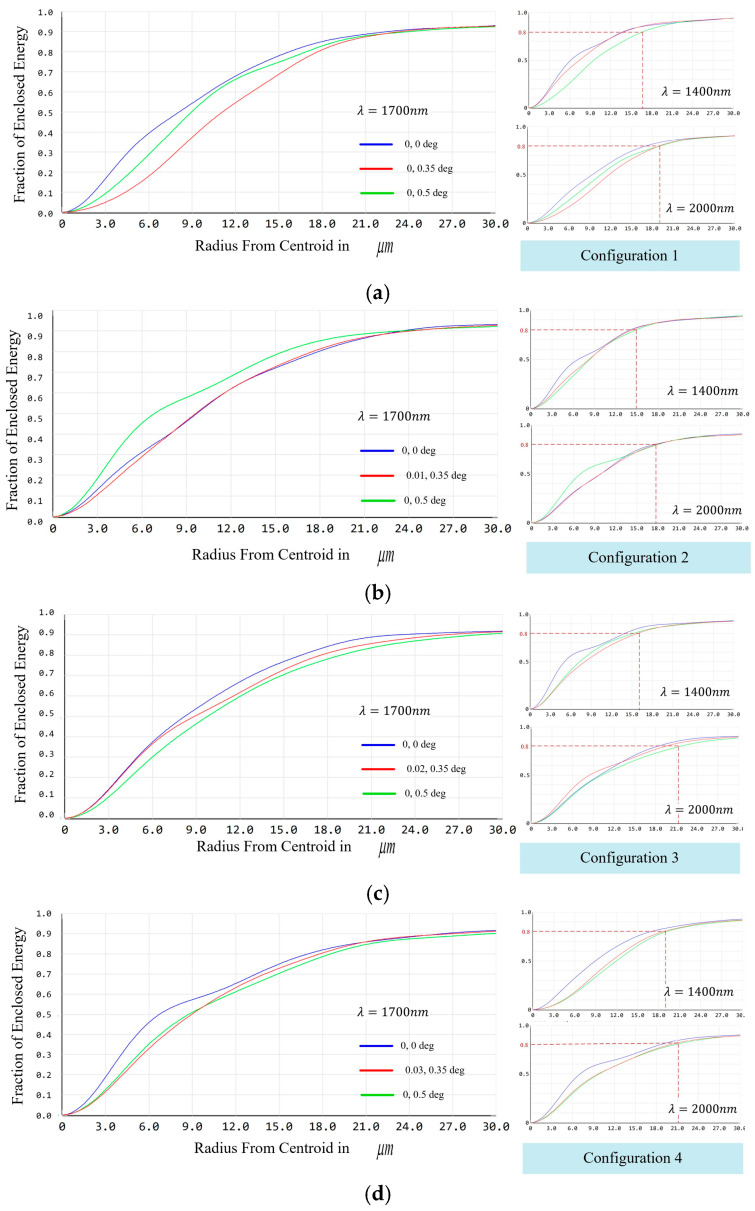
Energy concentration curves of the spectral imaging system based on an image-slicer IFU: (**a**) energy concentration curves in configuration 1 for the wavelengths of 1700 nm, 1400 nm, and 2000 nm; (**b**) energy concentration curves in configuration 2 for the wavelengths of 1700 nm, 1400 nm, and 2000 nm; (**c**) energy concentration curves in configuration 3 for the wavelengths of 1700 nm, 1400 nm, and 2000 nm; and (**d**) energy concentration curves in configuration 4 for the wavelengths of 1700 nm, 1400 nm, and 2000 nm.

Figure 10 shows the energy concentration curves for the center and edge wavelengths of each configuration, with 80% of the energy at the imaging point concentrated in the ∅24 μm range.

**Figure 11:**
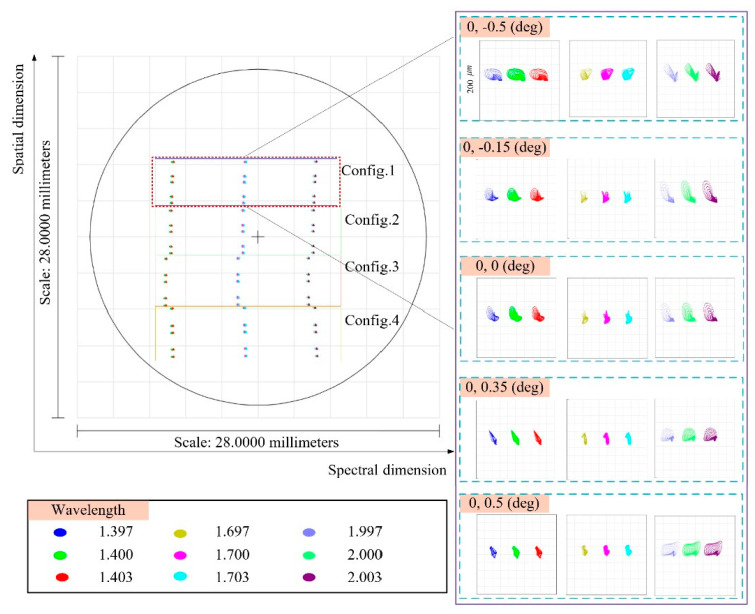
Footprint diagram in the image plane of the entire system and the spot diagram in configuration 1 for different wavelengths.

Figure 11 shows the image plane dispersion of the four sets of sub-slits and the spectral resolution maps of each sub-slit, with no light crosstalk between the sliced sub-fields of view. Taking the central FOV as an example, the spectral resolution of the integrated spectral imaging system is better than 3 nm.

The above design results show that adopting the idea of reducing the magnification step by step for the design and adding free-form surfaces for the overall optimization can make each configuration achieve good image quality in the whole band at the same time. This design enables the full FOV of a single slice of the IFU to be expanded, thus meeting the requirements of remote sensing.

## 5. Discussion and Conclusions

In general, traditional grating-type spectral imaging systems are unable to continuously collect spectral information of targets with transient variations due to the limitations of slits or pinholes, resulting in a limited FOV utilization of the telescope system. To address this issue, we developed a spectral imaging system based on an image slicer IFU. The system realized the expansion of the IFOV of the spectral imaging system by splitting the focal plane of the telescope system through the IFU, and corrected the aberrations caused by increasing the full FOV of a single slice of the IFU, realizing the conversion of the full FOV of a single slice of the IFU from angular arcseconds to degrees. Simulation results show that the system can capture targets within the spectral range of 1400 nm to 2000 nm with a spectral resolution exceeding 3 nm. Compared to a conventional grating-type spectral imaging system, the IFOV of the system is increased by a factor of four. In addition, it can capture all the spectral information of a target with transient changes in a single exposure, reducing system complexity and the dependence on platform stability.

The research work in this paper mainly focused on the differences in FOV and F-number between the remote-sensing field and astronomical field, given the design method of the image-slicer IFU spectral imaging system applicable to the remote-sensing field. And the step-by-step design and combination optimization of the whole system were carried out according to the index parameters. The results showed that the system has good image quality at each sub-slit, verifying the effectiveness and advantages of the system.

However, since the spectral range and spectral resolution belong to the same dimension of the area mapped on the detector, the present system involves a trade-off between spectral range and spectral resolution, which limits the detection efficiency of the spectral imaging system in the field of remote sensing for the Earth. Future work will focus on exploiting the ability of the image-slicer IFU to slice the focal plane to ensure that the IFOV is increased while extending the ability of its multiple slits to extend the spectral range, thus maximizing the advantages of the image-slicer IFU in the detection of dynamic remote-sensing targets. On this basis, we will attempt to improve the system configuration and develop an integrated spectral imaging system with a wide spectral range and high resolution.

## Figures and Tables

**Table 1:** Specifications of the spectral imaging system based on an image-slicer IFU.

Parameter	Value
Wavelength range	1400–2000 nm
Spatial resolution	<37.5 m
Spectral resolution	<3 nm
F-Number	4.7
Focal length	200 mm
IFOV	>0.5 mrad
Full FOV	1°
Slice number	4

**Table 2:** The tilt angles of each sub-mirror array.

Configuration	Slicer Mirror		Pupil Mirror	
	Tilt x/°	Tilt y/°	Tilt x/°	Tilt y/°
1	−13.000	−2.000	−21.075	2.572
2	−11.500	−4.000	−20.032	−3.503
3	−10.000	−2.000	−18.815	3.302
4	−8.500	−4.000	−17.745	−2.993

## Data Availability

The data presented in this study are available on request from the corresponding author. The data are not publicly available due to the data involves technical secrets.
